# In Vitro and In Silico Characterization of G-Protein Coupled Receptor (GPCR) Targets of Phlorofucofuroeckol-A and Dieckol

**DOI:** 10.3390/md19060326

**Published:** 2021-06-04

**Authors:** Pradeep Paudel, Su Hui Seong, Se Eun Park, Jong Hoon Ryu, Hyun Ah Jung, Jae Sue Choi

**Affiliations:** 1 Department of Food and Life Science, Pukyong National University, Busan 48513, Korea; ppradeep@olemiss.edu (P.P.); shseong@hnibr.re.kr (S.H.S.); gogo1685@mail.ulsan.ac.kr (S.E.P.); 2 National Center for Natural Products Research, Research Institute of Pharmaceutical Sciences, The University of Mississippi, Oxford, MS 38677, USA; 3 Natural Products Research Division, Honam National Institute of Biological Resource, Mokpo 58762, Korea; 4 Department of Biomedical Science, Asan Medical Institute of Convergence Science and Technology, Seoul 05505, Korea; 5 Department of Life and Nanopharmaceutical Science, Kyung Hee University, Seoul 02447, Korea; jhryu63@khu.ac.kr; 6 Department of Food Science and Human Nutrition, Jeonbuk National University, Jeonju 54896, Korea

**Keywords:** phhlorotannins, GPCRs, agonist, antagonist, dieckol, PFF-A, molecular docking

## Abstract

Phlorotannins are polyphenolic compounds in marine alga, especially the brown algae. Among numerous phlorotannins, dieckol and phlorofucofuroeckol-A (PFF-A) are the major ones and despite a wider biological activity profile, knowledge of the G protein-coupled receptor (GPCR) targets of these phlorotannins is lacking. This study explores prime GPCR targets of the two phlorotannins. In silico proteocheminformatics modeling predicted twenty major protein targets and in vitro functional assays showed a good agonist effect at the α2C adrenergic receptor (α_2C_AR) and an antagonist effect at the adenosine 2A receptor (A_2A_R), δ-opioid receptor (δ-OPR), glucagon-like peptide-1 receptor (GLP-1R), and 5-hydroxytryptamine 1A receptor (5-TH_1A_R) of both phlorotannins. Besides, dieckol showed an antagonist effect at the vasopressin 1A receptor (V_1A_R) and PFF-A showed a promising agonist effect at the cannabinoid 1 receptor and an antagonist effect at V_1A_R. In silico molecular docking simulation enabled us to investigate and identify distinct binding features of these phlorotannins to the target proteins. The docking results suggested that dieckol and PFF-A bind to the crystal structures of the proteins with good affinity involving key interacting amino acid residues comparable to reference ligands. Overall, the present study suggests α_2C_AR, A_2A_R, δ-OPR, GLP-1R, 5-TH_1A_R, CB_1_R, and V_1A_R as prime receptor targets of dieckol and PFF-A.

## 1. Introduction

G protein-coupled receptors (GPCRs) are a family of membrane receptors that regulate human pathophysiology and are the leading target class for pharmaceuticals. At present, GPCRs mediate the effect of approximately one-third of the FDA-approved drugs [1,2,3]. However, these drugs target mainly biogenic amine receptors, which comprise around 30 members of the GPCR family [3]. There is, therefore, an immense potential within pharmaceuticals/natural products to exploit, considering the remaining family members for which no existing ligands have been identified.

In the traditional drug development process, the high-throughput screening (HTS) approach against drug targets of choice is the very first step to uncover new drugs, which has now been augmented by the in silico method to maximize the probability of novel leads discovery. Traditional Chinese medicine (TCM) is an important research object of network (TCM herbs, targets, diseases, and syndromes) pharmacology, which aims to understand the network-based biological basis of complex diseases [4], and natural polyphenols are abundant in plant-based foods whose network proximity to disease proteins is predictive of the molecule’s known therapeutic effects [5].

Secondary metabolites from seaweeds have gained much interest in natural drug discovery, because the marine source is a huge reservoir of natural products with significant biological activities. In addition, secondary metabolites (carotenoids, polyphenols, and polysaccharides) with numerous biological activities make them a potential source of leads. Among marine organisms, marine alga, i.e., green algae (Chlorophyta), brown algae (Phaeophyta), and red algae (Rhodophyta), are rich sources of bioactive compounds with various biological activities. These macroalgae are well known by seaweeds and have been widely recognized as food, functional food, and potential drug sources for decades. Brown algae are the largest type of seaweed and so far, scientists have identified the therapeutic potential of brown algae-derived secondary metabolites (particularly phloroglucinol-based polyphenols, known as phlorotannins) including, but not limited to antioxidant [6,7], antimicrobial [8], anti-diabetic [9], anti-Alzheimer’s disease [10,11,12], anti-inflammatory [13], neuroprotective [14,15], anti-obesity [16], hepatoprotective [17], monoamine oxidase inhibitor [18], antihypertension [19] and anti-viral [20] activity. *Ecklonia stolonifera* OKAMURA (*E. stolonifera*) is an edible brown alga of the Laminariaceae family that is widely distributed along the Eastern and Southern Korean coast and rich in phlorotannins [19,21]. Dieckol and phlorofucofuroeckol-A (PFF-A) are common phlorotannins in *E. stolonifera* and in our recent study, we had reported human monoamine oxidase (hMAO) inhibition, dopamine D_3_R/D_4_R receptor agonist effect, dopamine D_1_/5-hydroxytryptamine 1A (5-HT_1A_)/neurokinin 1 (NK_1_) receptor antagonist effect [22], and β-secretase and acetylcholinesterase inhibition by dieckol and PFF-A [10,11]. Nonetheless, other promising targets of these phlorotannins are yet to be identified.

**Figure 1:**
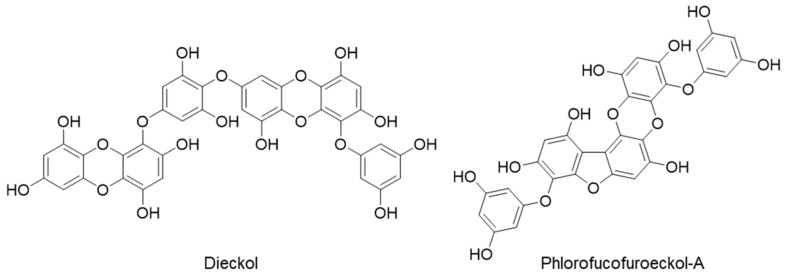
Chemical structures of dieckol and phlorofucofuroeckol-A.

Therefore, the main objectives of this study were to: (a) predict prime protein targets of dieckol and PFF-A (Figure 1) via proteocheminformatics modeling (PCM), (b) validate the PCM prediction by evaluating the modulatory effect on predicted receptors via cell-based functional GPCRs assays, and (c) look at the specific binding interactions of test ligands and target receptors via molecular docking simulation.

## 2. Results

### 2.1. In Silico Target Prediction

Proteocheminformatics (PCM) modeling is a quantitative bio-modeling technique that can predict the affinity and potency of a ligand against multiple different protein targets simultaneously by combining chemical and biological information from the ligand and related targets into a single machine learning model [23]. From in silico PCM modeling, the highest-ranked twenty potential protein targets were predicted for the phlorotannins. Table 1 presents a list of the target proteins with an average score value.

As shown in the Table 1, the V_1A_ receptor was predicted as a top target for dieckol and PFFA. For PFF-A, 5-hydroxytryptophan 1A (5-HT_1A_R), 5-hydroxytryptophan 1B (5-HT_1B_R), and cannabinoid 1 (CB_1_R) receptors were among the predicted top twenty protein targets. Based on this prediction and reported biological activities of the phlorotannins in the literature, we proceeded to validate adenosine A_2A_ receptor (A_2A_R), alpha-2A adrenergic receptor (α_2A_AR), alpha-2C adrenergic receptor (α_2C_AR), δ-opioid receptor (δ-OPR), CB_1_R, free fatty acid receptor 1 (FFA_1_R or GPR40), glucagon-like peptide-1 receptor (GLP-1), V_1A_R, 5-HT_1A_R, and 5-HT_1B_R cell-based functional assays.

Firstly, the functional effect of dieckol and PFF-A was screened at a 100-µM concentration. As shown in Table 2, dieckol showed an agonist effect on α_2C_AR (52.4 ± 4.24%) and V_1A_R (106.73 ± 2.97%) and an antagonist effect on A_2A_R (55.55 ± 4.03%), δ-OP (66.95 ± 0.92), CB_1_R (158.75 ± 17.81%), and GLP-1R (101.0 ± 8.20%).

Likewise, PFF-A showed an agonist effect on α_2C_AR (83.8 ± 0.07%) and CB_1_R (113.8 ± 3.68%) and an antagonist effect on A_2A_R (66.6 ± 2.26%), δ-OP (73.55 ± 5.44), and GLP-1R (105.7 ± 1.27%). These phlorotannins were either mild active or inactive at other tested protein targets as depicted by the negative and/or low value of % stimulation or inhibition (Table 2).

**Figure 2:**
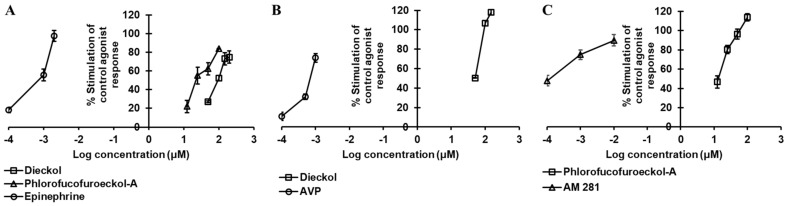
Dose-dependent agonist effect of dieckol and/or phlorofucofuroeckol-A on hα_2__C_AR (**A**), hV_1A_R (**B**), and hCB_1_ (**C**) receptors.

**Figure 3:**
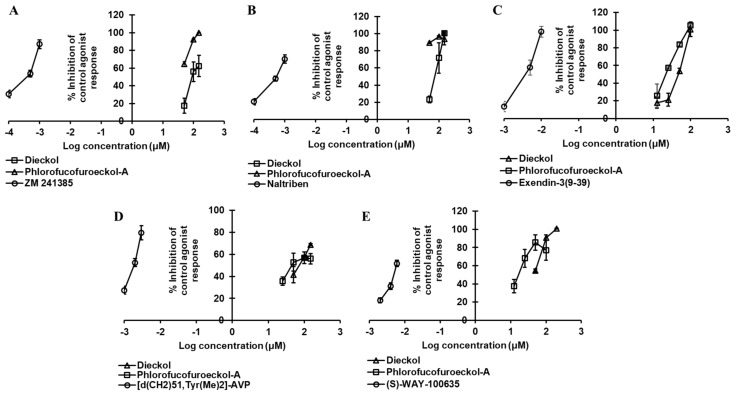
Dose-dependent antagonist effect of dieckol and phlorofucofuroeckol-A against hA_2A_ (**A**), δ-opioid (hδ-OP) (**B**), hGLP-1 (**C**), hV_1A_R (**D**), and h5-HT_1A_R (**E**) receptors.

Based on the functional effect above 50% at 100 µM, the concentration-dependent effect was further tested and compared with the reference agonists and antagonists (Figure 2 and Figure 3 and Table 3 and Table 4) followed by molecular docking simulation. Molecular docking simulation of test ligands to the crystal structures of target proteins and comparison with the reference ligands results revealed the mechanism of ligand–target-protein interaction.

### 2.2. Dieckol and PFF-A as A_2A_R Antagonists

Dieckol inhibited the 3 nM epinephrine bitartrate response by 17.5%, 56.0%, and 62.43% at a concentration of 50, 100, and 150 µM, respectively, and yielded an IC_50_ value of 87.18 ± 2.63 µM (Table 3 and Figure 3A), while PFF-A inhibited the response of the reference agonist by 64.7%, 92.2%, and 99.93% at a concentration of 50, 100, and 150 µM, yielding an IC_50_ value < 50 µM (Figure 3A).

**Figure 4:**
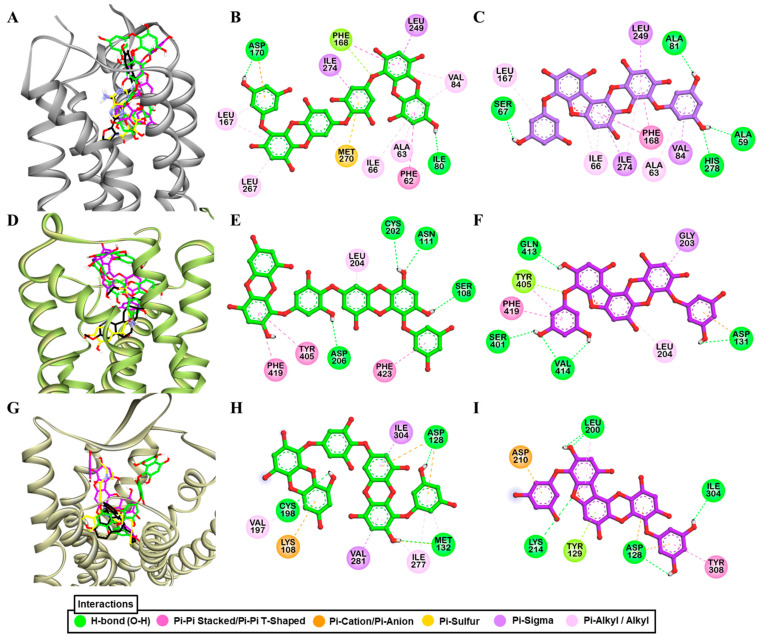
Molecular docking of dieckol and phlorofucofuroeckol-A in the active site of hA_2A_R (**A**), hα_2C_AR (**D**), and hδ-OPR (**G**) along with reported agonist (yellow stick) and antagonist (black stick). Detailed hA_2A_R–ligand (**B**) for dieckol and (**C**) for phlorofucofuroeckol-A), hα_2C_AR–ligand (**E**) for dieckol and (**F**) for phlorofucofuroeckol-A), and hδ-OPR–ligand interactions (**H**) for dieckol and (**I**) for phlorofucofuroeckol-A) on a 2D diagram.

In the docking simulation, dieckol formed two H-bond interactions with Ile80 and Asp170 (Figure 4B) while four H-bond interactions (His278, Ala59, Ala81, Ser67) were observed for PFF-A (Figure 3C). The binding of reference ligands to the A_2A_R crystal structure showed the involvement of residues Phe168, Leu249, Asn253, and Met270. The total number of hydrophobic and electrostatic interactions involved in dieckol binding was greater than that of PFF-A binding (Table S2). Interestingly, only one interacting residue (Leu249) was in common with the reference ligand. However, PFF-A had two common interacting residues (Leu249 and Phe168) with reference antagonist ZM241385 (Table S1).

### 2.3. Dieckol and PFF-A as α_2C_AR Agonists

Evaluation of the concentration-dependent agonist effect of phlorotannins (Table 3 and Figure 2A) at α_2C_AR depicted dieckol as a moderate agonist (EC_50_: 98.80 ± 7.71 µM) and PFF-A as a good agonist (EC_50_: 23.67 ± 3.32 µM). Even at a 25-µM concentration, PFF-A stimulated the effect of 1 µM epinephrine by 55%. The reference agonist epinephrine had an EC_50_ value of 0.86 nM. To further support the functional effect and delineate the difference in activity between the two phlorotannins, a molecular docking simulation of test ligands and target protein was performed.

As shown in Figure 4D,E, dieckol interacted with Asn111, Ser108, Cys202, Asp206, and Gly203 via H-bond (Figure 2B). Similarly, PFFA also displayed four H-bond interactions with Val414, Asp131, Ser401, and Gln413 (Figure 4F). H-bond interaction with Asp131 was a typical interaction observed for the reference agonist (epinephrine) and PFF-A, but absent in dieckol binding. Between two test ligands, hydrophobic interactions with Phe419, Tyr405, and Leu204 were common (Table S1).

### 2.4. Dieckol and PFF-A as δ-OPR Antagonists

The dose-dependent antagonist effect at the δ-opioid receptor depicted PFF-A as a potent natural antagonist. As shown in Table 4 and Figure 3B, even at the 50-µM concentration, PFF-A inhibited the effect of 25 nM [D—Pen2, D—Pen5]enkephalin (DPDPE) by 89.03 ± 0.70%, while the effect was 23.23 ± 4.04% for the same concentration of dieckol. Dieckol had an IC_50_ value of 80.46 ± 13.74 µM, but the value was <50 µM for PFF-A. The reference antagonist naltriben mesylate had an IC_50_ value of 9 nM. The binding of dieckol to the crystal structure of 4ej4 (Figure 4G,H) showed an involvement of three H-bond interactions (Asp128, Met132, Cys198) and numerous hydrophobic and electrostatic interactions (Met132 (Sulfur-O, π-alkyl), Lys108 (π-cation, π-Alkyl), Asp128 (π-anion), Val281 (π-sigma), Ile304 (π-sigma), Cys198 (π-sulfur), Ile277 (π-alkyl), and Val197 (π-alkyl)). Likewise, as shown in Figure 4I, PFF-A formed four H-bond interactions with Leu200, Lys214, Ile304, and Asp128 and five hydrophobic and electrostatic interactions with Asp128 (π-anion), Asp210 (π-Anion), Tyr129 (π-lone pair), Tyr308 (π-π stacked), and Leu200 (π-alkyl).

The reference antagonist naltrindole showed an H-bond interaction with aspartic acid residue (Asp128) and numerous hydrophobic interactions with tryptophan residues ‒ Trp284 (π-π-T-shaped), Trp284 (π-alkyl), and Trp274 (π-alkyl). Only Asp128 was a common interacting residue among the test and reference ligands while Tyr308 was observed for PFF-A and reference ligand binding, but not for dieckol (Tables S1 and S2).

### 2.5. PFF-A as a CB_1_R Agonist

**Figure 5:**
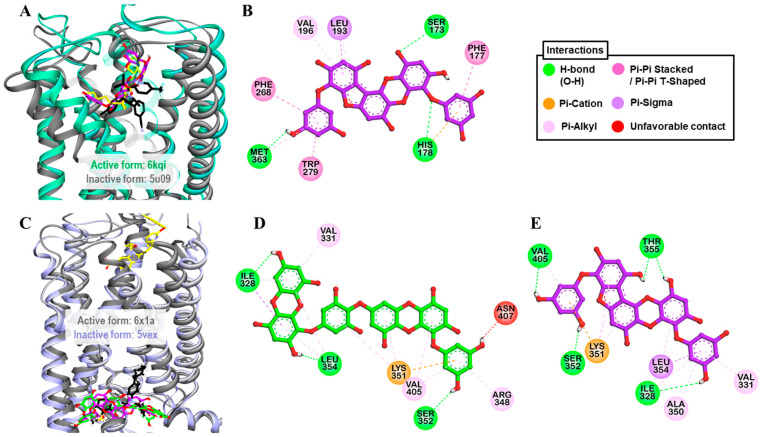
(**A**) Molecular docking of phlorofucofuroeckol-A (purple stick) in an active-state of *h*CB_1_R (PDB ID: 6kqi) along with reported agonist (yellow stick). Structure of reported antagonist taranabant docked into the inactive state of *h*CB_1_R (PDB ID: 5u09, gray ribbon) is shown as black stick. (**B**) Detailed *h*CB_1_R–ligand interactions on a 2D diagram for phlorofucofuroeckol-A. (**C**) Molecular docking of dieckol (green stick) and phlorofucofuroeckol-A (purple stick) in an inactive-state of *h*GLP-1 (PDB ID: 5vex, blue ribbon) along with reported antagonist, NNC0640 (black stick). Structure of reported agonist PF-06882961 docked into the active-state of *h*GLP-1 (PDB ID: 6x1a, gray ribbon) is shown as yellow stick. (**B**,**C**) Detailed *h*GLP-1–ligand interactions on a 2D diagram (**D**) for dieckol and (**E**) for phlorofucofuroeckol-A.

Only PFF-A showed a full CB_1_R agonist effect (113.8 ± 3.68%) at the 100-µM concentration. Therefore, the effect at lower concentrations was tested and, as shown in Table 3 and Figure 2C, PFF-A stimulated the effect of 10 nM CP 55940 by 46.7, 80.3, and 96.45% at 12.5, 25, and 50 µM, respectively. Hence, the log concentration vs. % simulation graph yielded an EC_50_ value of 13.42 ± 2.03 µM. Reference agonist CP 55940 had an EC_50_ value of 0.21 nM. To predict the binding affinity and characterize the binding mode of PFF-A and CB_1_R, molecular docking simulation was performed (Figure 5A). As tabulated in Tables S3 and S4, PFF-A interacted with the active-state CB_1_R (6kqi) by forming three H-bonds (Ser173, His178, and Met363) and numerous hydrophobic interactions—Phe177, Phe268, Trp279, Val196, Leu193, and Met363. Interactions with Ser173, Phe268, Phe177, Trp279, Val196, and Leu193 are a common observation in the binding of PFF-A and CP 55940 with the active-state CB_1_R (6kqi). The reference antagonist taranabant interacted with the inactive-state CB_1_R (5u09) by forming hydrogen-bond interactions with Ser173, Phe189, and Lys192 via the −CF_3_ group. Likewise, other hydrophobic interactions involved in taranabant–5u09 binding were phenylalanine residues (Phe170, Phe174, Phe189, Phe268, and Phe379), Trp279, His178, Leu192, Leu193, Ile267, and Met363 (Figure 5B).

### 2.6. Dieckol and PFF-A as GLP-1R Antagonists

Results from the functional assay on mouse GLP-1 receptor-expressed βTC6 cells demonstrated dieckol and PFF-A as full antagonists of the GLP-1 receptor. At a concentration of 100 μM, both the compounds inhibited the effect of 0.3 nM GLP-1(7–37) by 100%. However, at the 25-μM concentration, PFF-A inhibited the reference agonist-response by 57.37% and dieckol by 21.23%. Additionally, a dose-dependent response curve yielded IC_50_ values of 47.19 ± 2.46 and 21.56 ± 2.16 μM for dieckol and PFF-A, respectively (Table 4 and Figure 3C). The potency of PFF-A was two-fold higher than that of dieckol. The reference antagonist exendin-3(9–39) had an IC_50_ value of 4.6 nM. From the molecular docking study, hydrogen-bond interactions with Ser352 and Thr355 (Table S3) and hydrophobic interactions with Leu354, Lys351, and Val405 (Table S4) were common observations in test ligands and reference antagonist NNC0640 binding with an inactive-state GLP-1R (5vex) (Figure 5C–E) in our molecular docking simulation. An unfavorable contact between dieckol and GLP-1R receptor was observed via the Asn407 residue.

### 2.7. Dieckol as Agonist and PFF-A as Antagonist of hV_1A_R 

The agonist effect of dieckol at V_1A_R was first tested at 100 µM to compare with the effect of PFF-A that we reported earlier [22]. As tabulated in Table 2, dieckol at 100-µM concentration stimulated the percentage agonist effect of 1 μM arginine vasopressin (AVP) by 106.73 ± 2.97% and inhibited the percentage of control agonist response by 57.77 ± 0.32%. In the hV_1A_R antagonist assay, the 100-µM concentration of dieckol induced at least a 25% agonist effect. In comparison, PFF-A induced a 38.45 ± 7.14% stimulation and 56.90 ± 5.37% inhibition of the control agonist response at 100 µM. Furthermore, the concentration-dependent dose–response curve depicted dieckol as a hV_1A_R agonist (EC_50_: 39.12 ± 2.12 μM) (Table 3 and Figure 2B) and PFF-A as an antagonist (IC_50_: 42.25 ± 0.41 μM) (Table 4 and Figure 3D).

**Figure 6:**
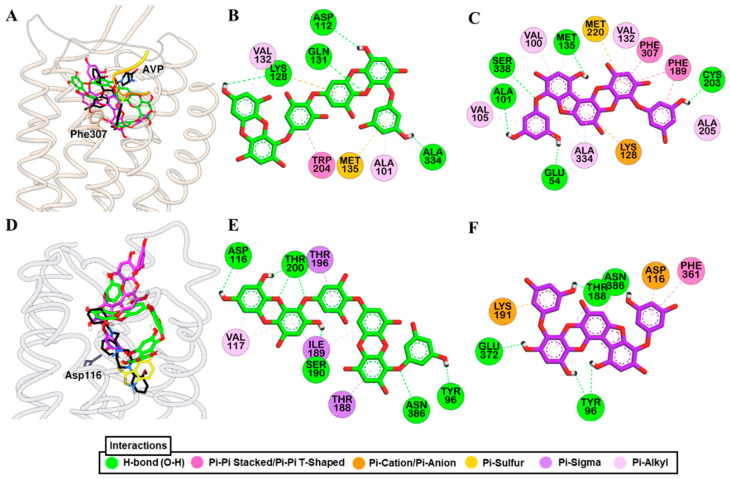
Molecular docking simulation of hV_1A_R (**A**) and h5-HT_1A_R (**D**) binding with dieckol (green stick) and phlorofucofuroeckol-A (purple stick) along with reported agonist (yellow ribbon or stick) and antagonist (black stick). Detailed hV_1A_R–ligand (**B**) for dieckol and (**C**) for phlorofucofuroeckol-A) and h5-HT_1A_R–ligand interactions (**E**) for dieckol and (**F**) for phlorofucofuroeckol-A) on a 2D diagram.

Molecular simulation of dieckol and PFF-A along with reference ligands to a crystal structure of hV_1A_R predicted that both test ligands bind with high affinity (Figure 6A). Dieckol formed H-bond interactions with Gln131, Ala334, and Asp112, and hydrophobic interactions with Lys128, Met135, Trp204, Ala101, and Ala334 (Figure 6B). Reference agonist AVP formed H-bond interactions with Asp202 (Salt-bridge), Glu54, Asp112, and Ile330 and hydrophobic interactions with Trp204, Ile330, Ala101, Ala334, Val132, and Met135. This shows that dieckol and AVP have numerous residues in common that involve binding with the receptor.

Likewise, PFF-A bound to the hV_1A_R via five H-bond interactions (Ser338, Cys203, Met135, Glu54, Ala101) and other hydrophobic interactions with Lys128, Met220, Phe189, Phe307, Val132, Val100, Ala101, Met135, Ala334, Ala205, and Val105 (Figure 6C). Three H-bond interactions with Gln131, Gln108, and Lys128, and hydrophobic interactions with Phe307, Trp204, Val132, Met135, Met220, Ala334, Ala205, Gln131, and Thr333 were observed for SR49059 binding. The docking result shows that, respective to their functional effect, dieckol and PFF-A interact with residues that were involved in the binding of the reference agonist and antagonist (Tables S5 and S6).

### 2.8. Dieckol and PFF-A as 5-HT_1A_R Antagonists

An antagonist effect was observed for dieckol and PFF-A in a cell-based functional assay. At 100-µM concentration, dieckol and PFF-A inhibited the response of 30 nM serotonin by 91.0 ± 3.11% and 77.00 ± 11.03%, respectively (Table 2). A concentration-dependent dose–response showed that dieckol and PFF-A inhibited the 50% response of 30 nM serotonin at 43.31 ± 3.22 and 17.75 ± 3.42 µM, respectively (Table 4 and Figure 3E). However, the agonist effect at 5-HT_1A_R was negligible for both the compounds when tested at the 100-µM concentration. As a result, the EC_50_ value was not determined. Reference drug serotonin had an EC_50_ value of 0.72 nM and antagonist GR55562 had an IC_50_ value of 4.4 nM.

Docking of test and reference ligands to the active site of 5-HT_1A_R demonstrated that aspartic acid residue Asp116 is one of the important binding residues (Figure 6D). Dieckol formed an H-bond interaction with Asp116, Thr200, Ser190, Asn386, and Tyr96 while PFF-A did with Thr188, Glu372, Tyr96, and Asn386 (Figure 6E,F). Reference ligands serotonin and WAY 100635 formed an H-bond interaction with Asp116 via a salt-bridge. Interactions with Thr200, Phe361, and Val117 were observed for test ligands and serotonin binding (Tables S5 and S6).

## 3. Discussion

Dieckol and PFF-A are phloroglucinol (1,3,5-trihydroxybenzene)-based polyphenols with a varied number of phloroglucinol units attached via dibenzofuran and dibenzodioxin linkages. Dieckol is a phloroglucinol hexamer and PFF-A is a phloroglucinol pentamer. A structure–activity relationship between phloroglucinol and its oligomers in our recent study [22] showed that more than three repeating phloroglucinol units are necessary for hMAOs inhibition and D_3_/D_4_ receptor agonist effect. Likewise, oligomerization of phloroglucinol with more than five repeating units is essential for the antagonist effect at D_1_, NK_1_, and 5-HT_1A_ receptors. Here, although the monomer phloroglucinol is not included in the study, the pentamer (PFF-A) showed better activity than a hexamer (dieckol). An interesting observation in this study is that regardless of the receptors at which these two phlorotannins showed functional effects (except the hV_1A_R), PFF-A was two-fold more potent than dieckol. In contrary to the findings that the phenolic -OH groups attached to the benzene ring of polyphenols play a vital role in the antioxidant effect [24,25,26] and that an increase in the number of hydroxyl groups increases antioxidant activity, the functional effect of PFF-A at tested GPCRs was higher than that of dieckol despite having a lower number of hydroxyl groups. The possible reason underlying this might be the structure or orientation of PFF-A that enables it to reach the core active site cavity of receptors where it binds to conserved interacting residues leading to conformational change.

Adenosine is an endogenous autacoid that regulates cellular physiology via adenosine A_1_, A_2A_, A_2B_, and A_3_ receptors. These receptors are expressed in several cells and tissues throughout the body and play a crucial role in regulating the pathophysiology of the human body, suggesting a potential drug target. Of different adenosine receptor subtypes, A_2A_R is the main receptor subtype in the striatum colocalized with dopamine D_2_ receptor and it modulates motor function [27,28]. Activation of A_2A_R decreases the binding affinity of D_2_R for agonists, implying A_2A_R antagonists as novel therapeutics for Parkinson’s disease [29]. At the synapse, A_2A_R facilitates glutamate release and potentiates NMDA receptor effects. It also stimulates glutamate release in astrocytes by inhibiting glutamate transporter-1 (GLT-1), and the level of A_2A_Rs in neurons and glia is significantly high in depression and Schizophrenia [30]. Hence, A_2A_Rs antagonists are effective as antidepressants and anti-anxiety agents. Here, dieckol and PFF-A showed an antagonist effect at hA_2A_R with IC_50_ values of 87.18 ± 2.63 and <50 µM, respectively. Furthermore, molecular docking simulation showed that dieckol and phlorofucofuroeckol-A strongly interact with the Phe168 residue, which is known as one of the important residues for ligand binding, via pi–pi interaction [31]. Structurally, dieckol and PFF-A are powerful radical scavengers [32] and as such, dieckol, in a recent study [33], protected dopaminergic neuronal cells by preventing α-synuclein aggregation via antioxidant mechanism. In a previous study [34], dieckol suppressed LPS-induced excessive microglial activation and protected neuronal cells by downregulating extracellular signal-regulated kinases, protein kinase B (PKB/Akt), and nicotinamide adenine dinucleotide phosphate hydrogen (NADPH) oxidase-mediated pathways. Likewise, PFF-A inhibited glutamate-induced apoptotic PC12 cell death in a caspase-dependent manner [35].

Similarly, at α_2C_AR, PFF-A showed a strong agonist effect and formed an H-bond with the Asp131 of α_2C_AR, which is the conserved active site residue. Adrenergic receptors are targets for epinephrine and norepinephrine and are involved in maintaining homeostasis. Among several types of adrenergic receptors, highly expressed α_2_ adrenoreceptors in astrocytes, and in glutamatergic and GABAergic neurons act by increasing intracellular Ca^2+^ levels [36]. The α_2C_AR subtype mediates cold-induced vasoconstriction, inhibits dopamine release in basal ganglia [37], and serotonin in the mouse hippocampus [38]. Therefore, α_2C_AR selective ligands have a therapeutic role in neuropsychiatric disorders [38] and α_2C_AR agonists are implicated in the treatment of neuropathic pain [39,40,41].

Serotonin (5-hydroxytryptamine, 5-HT) is a monoamine neurotransmitter that plays a crucial role in physiological functions, and of a total of 14 subtypes of 5-HT receptors, the 5-HT_1A_ receptor is a prominent target for the treatment of various neuropsychiatric and neurological disorders, prominently depression [42]. In the functional assay, dieckol and PFF-A showed a good antagonist effect at 5-HT_1A_R. Furthermore, they interacted with conserved aspartate residue (Asp116) of 5-HT_1A_R via H-bond and pi–anion binding, respectively.

Vasopressin is an antidiuretic hormone that plays a vital role in the central nervous system (CNS) and peripheral nervous system (PNS). The vasopressin receptor is one of the promising targets for CNS drugs, and vasopressin antagonists represent a novel approach for the treatment of stress, mood, and behavioral disorders [43]. Likewise, as a peripheral role, V_1A_R is responsible for vasoconstriction, myocardial contractility, platelet aggregation, and uterine contraction [44]. Similarly, in a recent study [45], upregulated vasopressin 1 receptor (V_1_R) expression in hepatocytes of ischemia-reperfusion injury mouse model was identified and the V_1_R/Wnt/β-catenin/FoxO3a/Akt pathway was highlighted as vital for hepatoprotection.

Cannabinoid CB_1_ receptors are among the most abundant GPCRs in the brain and they modulate CNS activity [46]. Cannabinoid CB_1_ receptor agonist activation of the CB_1_ receptor leads to decreased levels in cellular cAMP via inhibition of adenylyl cyclase. Moreover, CB_1_ activation inhibits voltage-gated Ca^2+^ channels and activates K^+^ channels, and these overall intracellular signaling activities reduce cellular excitability [47]. Likewise, studies also indicate high expression levels of CB_1_R in various types of cancer [48,49]. Interestingly, a new study demonstrated a higher orexigenic effect of the CB_1_R agonist AM11101 than tetrahydrocannabinol [50]. This shows that CB_1_R agonists could be used as an appetite stimulant in underweight patients. In the present study, only PFF-A showed a promising agonist effect at CB_1_R with an EC_50_ of 13.42 ± 2.03 µM. Several reports on PFF-A show neuroprotective effects mainly via antioxidant mechanisms [14,35,51]. Likewise, a recent study suggested the ATF3-mediated pathway as a possible mechanism of PFF-A-induced apoptosis in human colorectal cancer cells [52]. However, it remains unclear whether the neuroprotective and anticancer effect of PFF-A is via CB_1_R agonist activity.

The human CB_1_ receptor is an important therapeutic target for obesity and obsessive disorders and the mechanism of its transition state (either active or inactive) is vital for understanding the regulatory action of the receptor [53]. A salt bridge between conserved Asp-Arg-Tyr (DRY) motif in the C-terminal region of transmembrane 3 (TM3) and transmembrane 6 (TM6) characterizes the active or inactive conformation of the rhodopsin-like GPCRs [54]. In an inactive conformation of CB_1_R, TM6 packs against TM3 and transmembrane 5 (TM5) and G protein-interacting residues—Phe200 (helix III) and Trp356 (helix VI) are obstructed [55]. The reference inverse agonist (taranabant) is bound to the inactive state crystal structure by forming an H-bond interaction between the NH of taranabant and the hydroxyl of Ser383 and the −CF_3_ group with Ser173, Phe189, and Lys192. This result corroborates the findings of a previous study [56] which concluded that a strong H-bond between the -NH group of taranabant and the hydroxyl of Ser383 was vital for superior affinity to CB_1_R. Likewise, the agonist CP55940 formed π–π interactions with Phe170 and Phe268, and two H-bond interactions with Ser173 and Ser383 in a similar fashion, as reported earlier [56]. PFFA also formed a stable pi–pi interaction with Phe268 and an H-bond interaction with Ser173 of the active state crystal structure (6kqi), which could explain the agonist potency of PFFA in vitro.

Among the tested protein targets, CB_1_R, GLP-1, and GPR40 are obesity/T2DM related GPCRs and in the functional assays, PFF-A showed a good agonist effect at CB_1_R, while both the dieckol and PFF-A showed an antagonist effect at the GLP-1 receptor. Their effect at GPR40 was mild agonist. A gut-derived incretin hormone GLP-1 stimulates insulin and suppresses glucagon secretion, inhibits gastric emptying, and reduces appetite and food intake. In a previous study, intracerebroventricular injection of exendin (9–39), a specific GLP-1 antagonist, blocked the inhibitory effect of GLP-1 on food intake [57]. Hence, GLP-1 agonists represent a new class of antidiabetic agents [58]. In a recent study on the anti-diabetic effect in the zebrafish model [59], dieckol treatment reduced liver glucose-6-phosphate and phosphoenolpyruvate carboxykinase, and enhanced glucose transport and insulin sensitivity via protein kinase B (Akt) phosphorylation. It is of note that dieckol and PFF-A showed a good antagonist effect at GLP-1. Thus, the in vivo effects of these phlorotannins in GLP-1-mediated signaling are urgent.

In conclusion, the present study characterizes the receptors hA_2A_R, hα_2C_AR, hδ-OP, CB_1_R, GLP-1, hV_1A_R, and h5-HT_1A_R as prime protein targets of dieckol and PFF-A. Moreover, the binding mechanism of test ligands with the target proteins strengthens the study and warrants further in vivo studies.

## 4. Materials and Methods

### 4.1. Chemicals and Reagents

A transfected Chinese hamster ovary (CHO), Hela, a murine interleukin-3 dependent pro-B (Ba/F3), PC12, and rat basophil leukemia cell lines were obtained from Eurofins Scientific (Eurofins-Cerep, Le Bois I’Eveque, France). Buffers—Dulbecco’s modified Eagle medium (DMEM) buffer, 4-(2-hydroxyethyl)-1-piperazineethanesulfonic acid (HEPES) buffer, and Hank’s balanced salt solution (HBSS) buffer—were purchased from Invitrogen (Carlsbad, CA, USA). The reference agonists: 5′-*N*-ethylcarboxamidoadenosine (NECA), epinephrine bitartrate, epinephrine, DPDPE, CP 55940, linoleic acid, GLP-1(7–37, arginine vasopressin (AVP), and serotonin, and antagonists: ZM 241385, RX-821002, rauwolscine, naltriben mesylate, AM 281, exendin-3(9–39), [d(CH_2_)_5_^1^, Tyr (Me)_2_]-AVP, (S)-WAY-100635, and GR55562) were obtained from Sigma-Aldrich (St. Louis, MO, USA). All other chemicals and reagents purchased from Merck and Fluka were of the highest available grade unless otherwise stated.

### 4.2. Isolation of Phlorotannins

Phlorotannins—dieckol and PFF-A were isolated from the ethyl acetate fraction of *E. stolonifera* ethanolic extract, as described previously [11,22].

### 4.3. In Silico Prediction of Targets

To predict potential protein targets for the phlorotannins, a proteocheminformatics modeling (PCM) in silico target prediction method was employed, as described recently [60]. For full information on the model, readers are further directed to a previous report [61].

### 4.4. Functional GPCR Assay

The functional assay using transfected cells expressing human cloned receptors, PC12 cells for adenosine A_2A_ receptor, rat basophil leukemia cells for human adrenergic alpha2A receptor, human delta opioid (δ-OP) receptor, CHO cells for adrenergic alpha_2C_ receptor, human cannabinoid CB_1_, and vasopressin (V_1A_R), human embryonic kidney 293 (HEK-293) cells for free fatty acid receptor 1 (FFA_1_R or GPR40), βTC6 cells for the glucagon-like peptide-1 receptor (GLP-1), Ba/F3 cells for serotonin (5-HT_1A_), and Hela for 5-HT_1B_ receptors were carried out at Eurofins laboratory (Eurofins-Cerep, Le Bois I’Eveque, France). The in-house assay protocol and experimental conditions are reported in our previous reports [15,22,62]. The functional effect of dieckol and PFF-A was characterized based on their modulation effect on cytosolic Ca^2+^ ion mobilization using a fluorimetric detection method or by measuring their effect on cAMP modulation using homogeneous time-resolved fluorescence (HTRF) detection.

### 4.5. Measurement of cAMP Level

Functional activity of phlorotannins over hA_2A_R, hα_2C_AR, hCB_1_R, GLP-1R, and h5-HT_1B_R was determined by measuring their effects on cAMP production by the HTRF detection method using transected cells expressing human cloned receptors.

#### 4.5.1. Functional Activity over hA_2A_R

In brief, the PC12 cells were suspended in HBSS buffer (Invitrogen) complemented with 20 mM HEPES (pH 7.4), 0.2 U/mL ADA, and 100 µM rolipram, then distributed in microplates at a density of 2.10^3^ cells/well and preincubated for 5 min at room temperature (RT) in the presence of HBSS (basal control), the test compound, or the reference agonist or antagonist. For stimulated control measurement, separate assay wells contained 3 µM NECA. Following 10 min incubation at RT, the cells were lysed and the fluorescence acceptor (D2-labeled cAMP) and fluorescence donor (anti-cAMP antibody labeled with europium cryptate) were added. After 60 min at RT, the fluorescence transfer was measured at λex = 337 nm and λem = 620 and 665 nm using an EnVision microplate reader EnSpire (PerkinElmer, Waltham, MA, USA). The cAMP concentration was determined by dividing the signal measured at 665 nm by that measured at 620 nm (ratio). Agonist result was expressed as a percent of the control response to 3 µM NECA while the antagonist effect as percent inhibition of the control response to 100 nM NECA. The standard reference agonist was NECA and the antagonist was ZM 241385, which were tested in each experiment at several concentrations to generate a concentration–response curve from which their EC_50_ and IC_50_ values were calculated.

#### 4.5.2. Functional Activity over hα_2C_AR

Briefly, the transfected CHO cells suspended in HBSS buffer (Invitrogen) complemented with 20 mM HEPES (pH 7.4) and 500 µM IBMX were distributed in microplates at a density of 10^4^ cells/well in the presence of either of the following: For agonist assay—HBSS (basal control), epinephrine 1 µM (stimulated control) or various concentrations (EC_50_ determination), or the test compounds. For antagonist assay—HBSS (stimulated controls), rauwolscine 10 µM (basal control) or various concentrations (IC_50_ determination), or the test compounds. The reference agonist epinephrine and the adenylyl cyclase activator NKH 477 were added at respective final concentrations of 100 nM and 5 µM. For basal control measurements, epinephrine was omitted from the wells containing 3 µM rauwolscine. After 10 min at 37 °C, the cells were lysed and the fluorescence acceptor (D2-labeled cAMP) and fluorescence donor (anti-cAMP antibody labeled with europium cryptate) were added. After 60 min at RT, the fluorescence transfer was measured at λex = 337 nm and λem = 620 and 665 nm using a microplate reader (Envision, Perkin Elmer). The concentration of cAMP was determined by dividing the measured signal at 665 nm by that measured at 620 nm (ratio). The agonist result are shown as a percent of the control response to 1 µM epinephrine and the antagonist result are expressed as a percent inhibition of the control response to 30 nM epinephrine. Epinephrine and rauwolscine were the standard reference drugs used in each experiment at different concentrations.

#### 4.5.3. Functional Activity over hCB_1_R

The transfected CHO cells were suspended in HBSS buffer (Invitrogen) complemented with 20 mM HEPES (pH 7.4). Then, the cells were distributed in microplates at a density of 5.10^3^ cells/well in the presence of either of the following: For agonist assay—HBSS (basal control), 30 nM CP 55940 (stimulated control) or various concentrations (EC_50_ determination), or the test compounds. For antagonist assay—HBSS (stimulated controls), 10 µM AM 281 (basal control) or various concentrations for IC_50_ determination, or the test compounds. Thereafter, the reference agonist CP 55940 and the adenylyl cyclase activator forskolin were added at respective final concentrations of 1 nM and 25 µM. For basal control measurements, CP 55940 was excluded from the wells containing 10 µM AM 281. After 30 min of incubation at 37 °C, the cells were lysed and the fluorescence acceptor (D2-labeled cAMP) and fluorescence donor (anti-cAMP antibody labeled with europium cryptate) were added. The fluorescence transfer was measured at λex = 337 nm and λem = 620 and 665 nm using an Envision microplate reader (PerkinElmer, Waltham, MA, USA) after 60 min at RT. The agonist results are expressed as a percent of the control response to 10 nM CP 55940 and the antagonist results are expressed as percent inhibition of the control response to 1 nM CP 55940. CP 55940 and AM 281 were standard reference drugs that were tested in each experiment.

#### 4.5.4. Functional Activity over GLP-1R

The HBSS buffer (Invitrogen) complemented with 20 mM HEPES (pH 7.4) and 500 µM IBMX was used to suspend and distribute the βTC6 cells at a density of 1.5x10^4^ cells/well. The plate was then incubated for 10 min at RT in the presence of HBSS (basal and stimulated control), the test compound, or the reference agonist and antagonist. In the agonist assay, separate assay wells containing 100 nM GLP-1(7–37) were prepared for the stimulated control measurement, while in the antagonist assay, the reference agonist GLP-1(7–37) was added at a final concentration of 0.3 nM, and separate assay wells contained HBSS for basal control measurements. Following incubation, the cells were lysed and the fluorescence acceptor (D2-labeled cAMP) and fluorescence donor (anti-cAMP antibody labeled with europium cryptate) were added. After 60 min at room temperature, the fluorescence transfer was measured at λex = 337 nm and λem = 620 nm and 665 nm using an Envision microplate reader (PerkinElmer, Waltham, MA, USA). The results are expressed as either a percent of the control response to 100 nM GLP-1(7–37) or a percent inhibition of the control response to 0.3 nM GLP-1(7–37). The standard reference agonist was GLP-1(7–37) and the antagonist was exendin-3(9–39).

#### 4.5.5. Functional Activity over 5-HT_1B_R

Concisely, a plasmid containing the GPCR gene of interest (5-HT_1B_) was transfected into Hela cells. The resulting stable transfectants were suspended in HBSS buffer (Invitrogen, Carlsbad, CA, USA) containing 20 mM HEPES (pH 7.4), 400 mM NaCl, 1 mg/mL glucose, and 500 μM IBMX and distributed in microplates at a density of 2 × 10^4^ cells/well. The plates were then incubated for 20 min at RT in the presence of either of the following: HBSS and 0.1% BSA (basal control), serotonin at 10 μM (stimulated control) or various concentrations for EC_50_ determination, or the test phlorotannins. Thereafter, the adenylyl cyclase activator NKH 477 (5 μM) was added and the plates were incubated at 37 °C for 20 min. Then, the cells were lysed and a fluorescence acceptor (D2-labeled cAMP) and fluorescence donor (anti-cAMP antibody with europium cryptate) were added following 60 min incubation at RT. After incubation, the fluorescence transfer was measured using an Envision microplate reader (PerkinElmer, Waltham, MA, USA) and the results are expressed as a percentage of the control response to 10 µM serotonin for the agonist effect and as percent inhibition of the control response to 100 nM serotonin.

### 4.6. Measurement of Intracellular [Ca^2+^] Level

Functional activity of phlorotannins over human adrenergic α2A (hα_2A_), human δ-opioid (hδ-OP), free fatty acid receptor 1 (FFA_1_R/GPR40), human vasopressin 1A (hV_1A_), and human serotonin 1A (h5-HT_1A_) receptors was assessed by measuring their effect on cytosolic Ca^2+^ ion mobilization at the transected cells expressing human cloned receptors using a fluorimetric detection method.

#### 4.6.1. Functional Activity over hα_2A_AR

The rat basophil leukemia cells were distributed in microplates at a density of 1.1 × 10^4^ cells/well after suspending in a HBSS buffer (Invitrogen) containing 20 mM HEPES. Then, the fluorescent probe (Fluo8, AAT Bioquest) mixed with probenecid in HBSS buffer (Invitrogen) complemented with 20 mM Hepes (Millipore) (pH 7.4) was added into each well incubated for 60 min at 30 °C. Thereafter, the assay plates were positioned in a microplate reader (FlipR Tetra, Molecular Device) and we added test compounds, reference agonist/antagonist or HBSS buffer (basal control). Change in fluorescence intensity which varies proportionally to the free cytosolic Ca^2+^ ion concentration was measured. For stimulated control measurements, separate assay wells containing 0.1 µM epinephrine bitartrate were prepared. The agonist effect was calculated as a % of control response to epinephrine bitartrate at 0.1 µM. Similarly, for the antagonist effect, % inhibition of the control response to epinephrine bitartrate at 3 nM was evaluated. Epinephrine bitartrate and RX-821002 were used as reference agonists and antagonists, respectively.

#### 4.6.2. Functional Activity over hδ-OPR

At first, rat basophil leukemia cells were suspended in HBSS buffer (Invitrogen) complemented with 20 mM HEPES, and distributed in microplates at a density of 2.768 × 10^4^ cells/well. Thereafter, a mixture of fluorescent probe (Fluo8, AAT Bioquest) and probenecid in HBSS buffer (Invitrogen) complemented with 20 mM Hepes (Millipore) (pH 7.4) was added and plates were incubated for 60 min at 30 °C. Then, the assay plates were positioned in a FlipR Tetra microplate reader (Molecular Device, San Jose, CA, USA) for the addition of the test compound, reference agonist/antagonist, or HBSS buffer (basal control). Change in fluorescence intensity that varies proportionally to the free cytosolic Ca^2+^ ion concentration was measured.

For stimulated control measurements, 1 µM DPDPE was added in separate assay wells. The results are expressed as a percent of the control response to DPDPE at 1 µM or a percent inhibition of the control response to DPDPE at 25 nM. The standard reference agonist and antagonist were DPDPE and naltriben mesylate, respectively.

#### 4.6.3. Functional Activity over FFA_1_R/GPR40

In general, transfected HEK-293 cells suspended in DMEM buffer (Invitrogen) containing 1% FCSd were distributed in microplates at a density of 2.10^4^ cells/well. Then, the mixture of fluorescent probe (Fluo4 Direct, Invitrogen) and probenecid in HBSS buffer (Invitrogen) complemented with 20 mM Hepes (Invitrogen) (pH 7.4) was added into each well and incubated for 60 min at 37 °C, followed by 15 min incubation at 22 °C. Thereafter, the assay plates were positioned in a CellLux microplate reader (PerkinElmer, Waltham, MA, USA) which was used for the addition of the following: For agonist assay—test compound, reference agonist, or HBSS buffer (basal control). Linoleic acid at 100 µM was added in separate assay wells for stimulated control measurement. For antagonist assay—test compound or HBSS buffer (basal and stimulated control), then, 5 min later, 20 µM linoleic acid. Agonist results are expressed as a percent of the control response to 100 µM linoleic acid while antagonist results are expressed as percent inhibition of the control response to 20 µM linoleic acid.

#### 4.6.4. Functional Activity over hV_1A_R

Briefly, CHO-V_1A_R cells were separately suspended in DMEM buffer (Invitrogen, Carlsbad, CA, USA) complemented with 0.1% FCSd and distributed into microplates (4.5× 10^4^ cells/well). Then, fluorescent probe (Fluo4, Invitrogen) mixed with probenecid in HBSS buffer (Invitrogen, Carlsbad, CA, USA) supplemented with 20 mM HEPES, pH 7.4 (Invitrogen) was added to each well, allowing to equilibrate with the cells for 60 min at 37 °C, then 15 min at 22 °C. Thereafter, the assay plates were positioned in a CellLux microplate reader (PerkinElmer, Waltham, MA, USA) and dieckol and PFF-A (12.5, 25, 50, 100, and/or 150 μM), reference agonist, or HBSS buffer (basal control) was added. For stimulated control measurements, AVP at 1 µM was added in separate assay wells. The agonist effect on V_1A_R was calculated as a % of control response to 1 μM AVP. Similarly, for the antagonist effect, % inhibition of the control response to 10 nM AVP was evaluated. AVP and [d(CH_2_)_5_^1^, Tyr (Me)_2_]-AVP were used as reference agonist and antagonist, respectively.

#### 4.6.5. Functional Activity over h5-HT_1A_R

In brief, Ba/F3-5HT_1A_R cells were first suspended in HBSS buffer (Invitrogen, Carlsbad, CA, USA) complemented with 20 mM HEPES buffer (pH 7.4). Then, the cells were distributed into microplates at a density of 1 × 10^6^ cells/well. Subsequently, fluorescent probe (Fluo8, AAT Bioquest) mixed with probenecid in HBSS buffer (Invitrogen, Carlsbad, CA, USA) supplemented with 20 mM HEPES (Invitrogen) (pH 7.4) was added to each well, and the plates were incubated for 60 min at 37 °C. Thereafter, plates were fixed in a FlipR Tetra microplate reader (Molecular Device, San Jose, CA, USA) and dieckol and PFF-A (12.5, 25, 50, 100 and/or 150 μM), reference agonist, or HBSS buffer (basal control) was added. Fluorescence intensity was measured which varied in proportion to the free cytosolic Ca^2+^ ion concentration. Agonist effect on 5-HT_1A_R was calculated as a % of control response to 2.5 μM serotonin. Similarly, the percentage inhibition of the control response to 30 nM serotonin was calculated for the antagonist effect. Serotonin and (S)-WAY-100635 were used as reference agonists and antagonists, respectively.

### 4.7. Homology Modeling and Molecular Docking

The primary sequence of the human 5-HT_1A_R and human V_1A_R was obtained from UniProt (ID: P08908 and P37288, respectively). Based on the SWISS-MODEL, the 5-HT_1B_ receptor (PDB: 5V54) was selected as a template for homology modeling of human 5-HT_1A_ because it showed a good sequence similarity (0.42), sequence identity (42.97), and quaternary structure quality estimate (QSQE) (0.32) to this receptor. Similarly, µ-opioid receptor (PDB: 4DKL) was selected as a template for homology modeling of human V_1A_R, because it showed a good sequence similarity (0.32), sequence identity (24.54), and QSQE (0.19) to this receptor. The constructed model was refined using the ModRefiner server. Automated docking simulations were carried out with the AutoDock 4.2. program [63]. The structures of dieckol and PFF-A were generated and converted into 3D structures using Marvin Sketch (v17,1,30, ChemAxon, Budapest, Hungary). Structures of dieckol and PFF-A were energy-minimized using a molecular mechanics 2 (MM2) force field. X-ray crystallographic structures of GPCRs were obtained from the RCSB protein data bank (PDB) with respective PDB IDs—hA_2A_R (3eml) [31], hα_2C_AR (6kuw), hδ-OP (4ej4) [64], hCB_1_R (6kqi) [65], and hGLP-1 [66]. The structures of reported agonists (5′-*N*-ethylcarboxamidoadenosine (NECA), epinephrine, DPI-287, CP 55940, PF-06882961, AVP, and serotonin, and antagonists (ZM241385, RS-79948, naltrindole, taranabant, NNC0640, SR49059, and WAY 100635) were downloaded from PubChem or PDB. For each ligand–protein complex, 10 docking poses were generated using the same grid parameters (size and center) and docking parameters (genetic algorithm and run options). The pose for the lowest binding energy was chosen for the final docking result. When the root-mean-square deviation (RMSD) value between our docking result and the original crystallographic structures of the protein was less than 0.15 nm, we considered our docking protocol to be valid and performed the simulation. Results were analyzed and visualized using Discovery Studio (v17.2, Accelrys, San Diego, CA, USA).

## Figures and Tables

**Table 1:** List of top 20 protein targets from proteocheminformatics modeling (PCM) prediction of dieckol and phlorofucofuroeckol-A, respectively.

Rank	Dieckol		Phlorofucofuroeckol-A	
	Protein Name	Average Score	Protein Name	Average Score
1	Vasopressin 1A receptor	0.513	Vasopressin 1A receptor	0.797
2			Vasopressin 1B receptor	0.742
3			Oxytocin receptor	0.737
4			B2 bradykinin receptor	0.735
5			B1 bradykinin receptor	0.727
6			Histamine H1 receptor	0.721
7			Serotonin 1D receptor	0.717
8			Type-1 angiotensin II receptor	0.716
9			Dopamine D2 receptor	0.713
10			Cannabinoid receptor 1	0.711
11			Prostanoid EP3 receptor	0.710
12			Rho-associated protein kinase 1	0.710
13			Muscarinic acetylcholine receptor M3	0.709
14			Cholecystokinin A receptor	0.709
15			Serotonin 1A receptor	0.706
16			Neurokinin 1 receptor	0.706
17			Cysteinyl leukotriene receptor 1	0.706
18			Alpha-1D adrenergic receptor	0.705
19			Cholecystokinin B receptor	0.704
20			Serotonin 1B receptor	0.704

**Table 2:** Agonist and antagonist effect of 100 µM dieckol and phlorofucofuroeckol-A at several GPCRs.

GPCRs	Functional Effect at 100 µM Concentration			
	Dieckol		Phlorofucofuroeckol-A	
	Agonist Effect	Antagonist Effect	Agonist Effect	Antagonist Effect
Adenosine A2A receptor (A_2A_R)	−0.1 ± 1.41	55.55 ± 4.03	−0.7 ± 0.57	66.6 ± 2.26
Alpha-2A adrenergic receptor (α_2A_AR)	13.4 ± 19.87	46.15 ± 20.15	−0.5 ± 0.85	20.95 ± 1.77
Alpha-2C adrenergic receptor (α_2C_AR)	52.4 ± 4.24	−1.2 ± 6.08	83.8 ± 0.07	19.2 ± 9.76
δ-opioid receptor (δ-OPR)	−5.7 ± 0.14	66.95 ± 0.92	14.7 ± 7.35	73.55 ± 5.44
Cannabinoid receptor 1(CB_1_R)	−23.3 ± 12.09	158.75 ± 17.18	113.8 ± 3.68	21.35 ± 0.49
Free fatty acid receptor 1 (FFA1R) (GPR40)	0.2 ± 1.56	22.55 ± 5.44	−1.0 ± 0.07	30.15 ± 0.78
Glucagon-like peptide-1 receptor (GLP-1)	−16.3 ± 1.13	101 ± 8.20	−15.5 ± 2.55	105.7 ± 1.27
Vasopressin 1A receptor (V_1A_R)	106.73 ± 2.97	57.77 ± 0.32 ^b^	38.45 ± 7.14 ^a^	56.90 ± 5.37 ^b^
5-hydroxytryptophan 1A (5-HT_1A_R)	1.75 ± 0.64 ^a^	91.0 ± 3.11	1.65 ± 0.49 ^a^	77.00 ± 11.03
5-hydroxytryptophan 1B (5-HT_1B_R)		−7.3 ± 3.96		−18.5 ± 2.69

^a^ Value was extracted from our previous study [22]. ^b^ The test compound induces at least a 25% agonist effect at this concentration, which results in an apparent inhibition.

**Table 3:** Concentration-dependent agonist effect of dieckol and phlorofucofuroeckol-A at several GPCRs.

Compounds (µM)		Target GPCRs								
		hA_2A_R	hα_2A_AR	hα_2C_AR	hδ-OPR	CB_1_R	GPR40	GLP-1	hV_1A_R	h5-HT_1A_R
Dieckol	12.5	*–*	*–*	*–*	*–*	*–*	*–*	*–*	*–*	*–*
	25	*–*	*–*	*–*	*–*	*–*	*–*	*–*	*–*	*–*
	50	*–*	*–*	*–*	*–*	*–*	*–*	*–*	50.40 ± 0.42	*–*
	100	−0.1 ± 1.41	13.4 ± 19.87	52.4 ± 4.24	−5.7 ± 0.14	−23.3 ± 12.09	0.2 ± 1.56	−16.3 ± 1.13	106.73 ± 2.97	1.75 ± 0.64 ^c^
	150	*–*	*–*	73.0 ± 6.42	*–*	*–*	*–*	*–*	118.1 ± 2.83	*–*
	200	*–*	*–*	74.77 ± 6.60	*–*	*–*	*–*	*–*	*–*	*–*
EC_50_ (µM) ^a^		*NA*	*NA*	98.80 ± 7.71	*NA*	*NA*	*NA*	*NA*	39.12 ± 2.12	*NA*
PFF-A	12.5	*–*	*–*	22.03 ± 6.61	*–*	46.7 ± 6.22	*–*	*–*	*–*	*–*
	25	*–*	*–*	55.0 ± 9.13	*–*	80.3 ± 4.10	*–*	*–*	*–*	*–*
	50	*–*	*–*	62.27 ± 6.53	*–*	96.45 ± 5.02	*–*	*–*	*–*	*–*
	100	−0.7 ± 0.57	−0.5 ± 0.85	83.8 ± 0.07	14.7 ± 7.35	113.8 ± 3.68	−1.0 ± 0.07	−15.5 ± 2.55	38.45 ± 7.14 ^c^	1.65 ± 0.49 ^c^
	150	*–*	*–*	*–*	*–*	*–*	*–*	*–*	*–*	*–*
	200	*–*	*–*	*–*	*–*	*–*	*–*	*–*	*–*	*–*
EC_50_ (µM) ^a^		*NA*	*NA*	23.67 ± 3.32	*NA*	13.42 ± 2.03	*NA*	*NA*	*NA*	*NA*
Reference Drugs, EC_50_ (nM) ^b^		9.1	0.74	0.86	4.4	0.21	10000	0.049	0.72	2.5 ^c^

^a^ The 50% effective concentration (EC_50_) values of compounds were expressed as mean ± SD, n = 3. ^b^ EC_50_ values of reference drugs (hA_2A_R: 5′-N-ethylcarboxamidoadenosine (NECA), hα_2A_AR: epinephrine bitartrate, hα_2C_AR: epinephrine, hδ-OPR: DPDPE, CB_1_R: CP 55940, GPR40: linoleic acid, GLP−1: GLP-1(7-37), hV_1A_R: AVP, h5-HT_1A_R: serotonin, h5-HT_1B_R: serotonin). ^c^ Value was extracted from our previous study [22]. *NA* No activity. *(–)* Not tested.

**Table 4:** Concentration-dependent antagonist effects of dieckol and phlorofucofuroeckol-A at several GPCRs.

Compounds (µM)		Target GPCRs									
		hA_2A_R	hα_2A_AR	hα_2C_AR	hδ-OPR	CB_1_R	GPR40	GLP-1	hV_1A_R	h5-HT_1A_R	h5-HT_1B_R
Dieckol	12.5	*–*	*–*	*–*	*–*	*–*	*–*	17.87 ± 6.32	*–*	*–*	*–*
	25	*–*	*–*	*–*	*–*	*–*	*–*	21.23 ± 7.31	–	*–*	*–*
	50	17.5 ± 8.50	*–*	*–*	23.23 ± 4.04	*–*	*–*	53.80 ± 3.12	41.53 ± 7.39 ^c^	54.7 ± 1.7	*–*
	100	56.0 ± 11.11	46.15 ± 20.15	−1.20 ± 6.08	71.6 ± 17.85	−1.20 ± 6.08	22.55 ± 5.44	101.0 ± 8.20	57.77 ± 0.32 ^c^	91.0 ± 3.11	−7.3 ± 3.96
	150	62.43 ± 12.19	*–*	*–*	100.1 ± 1.53	*–*	*–*	*–*	68.80 ± 0.85 ^c^	*–*	*–*
	200	*–*	*–*	*–*	*–*	*–*	*–*	*–*	*–*	100.9 ± 0.57	−8.1 ± 0.85
IC_50_ (µM) ^a^		87.18 ± 2.63	*NA*	*NA*	80.46 ± 13.74	*NA*	*NA*	47.19 ± 2.46	82.71 ± 8.73	43.31 ± 3.22	*NA*
PFF-A	12.5	*–*	*–*	*–*	*–*	*–*	*–*	25.63 ± 13.14	*–*	37.40 ± 2.19	*–*
	25	*–*	*–*	*–*	*–*	*–*	*–*	57.37 ± 1.15	35.67 ± 3.88	68.20 ± 9.89	*–*
	50	64.7 ± 2.72	*–*	*–*	89.03 ± 0.70	*–*	*–*	83.87 ± 2.03	52.80 ± 8.09	85.55 ± 8.41	*–*
	100	92.2 ± 0.95	20.95 ± 1.77	19.2 ± 9.76	96.47 ± 0.84	21.35 ± 0.49	30.15 ± 0.78	105.7 ± 1.27	56.90 ± 5.37 ^c^	77.00 ± 11.03	−18.5 ± 2.69
	150	99.93 ± 0.31	*–*	*–*	93.67 ± 6.67	*–*	*–*	*–*	56.07 ± 4.72	–	*–*
	200	*–*	*–*	*–*	*–*	*–*	*–*	*–*	*–*	*–*	−35.5 ± 4.38
IC_50_ (µM) ^a^		< 50	*NA*	*NA*	< 50	*NA*	*NA*	21.56 ± 2.16	42.25 ± 0.41	17.75 ± 3.42	*NA*
Reference Drugs, IC_50_ (nM) ^b^		0.41	17	22	9	77	*ND*	4.6	1.9	4.4	23

^a^ The 50% inhibition concentration (IC_50_) values of compounds were expressed as mean ± SD, n = 3. ^b^ IC_50_ values of reference drugs (hA_2A_R: ZM 241385, hα_2A_AR: RX-821002, hα_2C_AR: rauwolscine, hδ-OPR: naltriben mesylate, CB_1_R: AM 281, GLP-1: exendin-3(9–39), hV_1A_R: [d(CH_2_)_5_^1^,Tyr(Me)_2_]-AVP, h5-HT_1A_R: (S)-WAY-100635, h5-HT_1B_R: GR55562). ^c^ The test compound induces at least 25% agonist effect at this concentration, which results in an apparent inhibition. *NA* No activity. *(–)* Not tested.

## Supplementary Materials

The following are available online at https://www.mdpi.com/article/10.3390/md19060326/s1, Table S1: Hydrogen bonding interaction residues between ligand–GPCRs (hA_2A_R, hα_2C_AR, and hDOP), Table S2: Hydrophobic and electrostatic interaction residues between ligand–GPCRs (hA_2A_R, hα_2C_AR, and hDOP), Table S3: Hydrogen, halogen, or electrostatic bonding interaction residues between ligand–GPCRs (hCB_1_R and hGLP-1), Table S4: Hydrophobic interaction residues between ligand–GPCRs (hCB_1_R and hGLP-1), Table S5: Hydrogen bonding interaction residues between ligand–GPCRs (hV_1A_R and h5-HT_1A_R), and Table S6: Hydrophobic and electrostatic interaction residues between ligand–GPCRs (hV_1A_R and h5-HT_1A_R).

## References

[1] Iliopoulos-Tsoutsouvas C., Kulkarni R.N., Makriyannis A., Nikas S.P. (2018). Fluorescent probes for G-protein-coupled receptor drug discovery. Expert Opin. Drug Discov..

[2] Schlyer S., Horuk R. (2006). I want a new drug: G-protein-coupled receptors in drug development. Drug Discov. Today.

[3] Kim H.R., Xu J., Maeda S., Duc N.M., Ahn D., Du Y., Chung K.Y. (2020). Structural mechanism underlying primary and secondary coupling between GPCRs and the Gi/o family. Nat. Commun..

[4] Wang X., Wang Z.-Y., Zheng J.-H., Li S. (2021). TCM network pharmacology: A new trend towards combining computational, experimental and clinical approaches. Chin. J. Nat. Med..

[5] Do Valle I.F., Roweth H.G., Malloy M.W., Moco S., Barron D., Battinelli E., Loscalzo J., Barabási A.-L. (2021). Network medicine framework shows that proximity of polyphenol targets and disease proteins predicts therapeutic effects of polyphenols. Nat. Food.

[6] Nakamura T., Nagayama K., Uchida K., Tanaka R. (1996). Antioxidant activity of phlorotannins isolated from the brown alga *Eisenia bicyclis*. Fish. Sci..

[7] Manandhar B., Wagle A., Seong S.H., Paudel P., Kim H.-R., Jung H.A., Choi J.S. (2019). Phlorotannins with potential anti-tyrosinase and antioxidant activity isolated from the marine seaweed *Ecklonia stolonifera*. Antioxidants.

[8] Eom S.-H., Kim Y.-M., Kim S.-K. (2012). Antimicrobial effect of phlorotannins from marine brown algae. Food Chem. Toxicol..

[9] Lee S.-H., Jeon Y.-J. (2013). Anti-diabetic effects of brown algae derived phlorotannins, marine polyphenols through diverse mechanisms. Fitoterapia.

[10] Jung H.A., Oh S.H., Choi J.S. (2010). Molecular docking studies of phlorotannins from *Eisenia bicyclis* with BACE1 inhibitory activity. Bioorg. Med. Chem. Lett..

[11] Yoon N.Y., Chung H.Y., Kim H.R., Choi J.E. (2008). Acetyl- and butyrylcholinesterase inhibitory activities of sterols and phlorotannins from *Ecklonia stolonifera*. Fish. Sci..

[12] Seong S.H., Paudel P., Jung H.A., Choi J.S. (2019). Identifying phlorofucofuroeckol-A as a dual inhibitor of amyloid-β25-35 self-aggregation and insulin glycation: Elucidation of the molecular mechanism of action. Mar. Drugs.

[13] Jung H.A., Jin S.E., Ahn B.R., Lee C.M., Choi J.S. (2013). Anti-inflammatory activity of edible brown alga *Eisenia bicyclis* and its constituents fucosterol and phlorotannins in LPS-stimulated RAW264.7 macrophages. Food Chem. Toxicol..

[14] Ahn B.R., Moon H.E., Kim H.R., Jung H.A., Choi J.S. (2012). Neuroprotective effect of edible brown alga *Eisenia bicyclis* on amyloid beta peptide-induced toxicity in PC12 cells. Arch. Pharm. Res..

[15] Paudel P., Seong S.H., Wu S., Park S., Jung H.A., Choi J.S. (2019). Eckol as a potential therapeutic against neurodegenerative diseases targeting dopamine D3/D4 receptors. Mar. Drugs.

[16] Jung H.A., Jung H.J., Jeong H.Y., Kwon H.J., Ali M.Y., Choi J.S. (2014). Phlorotannins isolated from the edible brown alga *Ecklonia stolonifera* exert anti-adipogenic activity on 3T3-L1 adipocytes by downregulating C/EBPα and PPARγ. Fitoterapia.

[17] Lee M.-S., Shin T., Utsuki T., Choi J.-S., Byun D.-S., Kim H.-R. (2012). Isolation and identification of phlorotannins from *Ecklonia stolonifera* with antioxidant and hepatoprotective properties in tacrine-treated HepG2 cells. J. Agri. Food Chem..

[18] Jung H.A., Roy A., Jung J.H., Choi J.S. (2017). Evaluation of the inhibitory effects of eckol and dieckol isolated from edible brown alga *Eisenia bicyclis* on human monoamine oxidases A and B. Arch. Pharm. Res..

[19] Jung H.A., Hyun S.K., Kim H.R., Choi J.S. (2006). Angiotensin-converting enzyme I inhibitory activity of phlorotannins from *Ecklonia stolonifera*. Fish. Sci..

[20] Kwon H.-J., Ryu Y.B., Kim Y.-M., Song N., Kim C.Y., Rho M.-C., Jeong J.-H., Cho K.-O., Lee W.S., Park S.-J. (2013). In vitro antiviral activity of phlorotannins isolated from *Ecklonia cava* against porcine epidemic diarrhea coronavirus infection and hemagglutination. Bioorg. Med. Chem..

[21] Kang H.S., Chung H.Y., Kim J.Y., Son B.W., Jung H.A., Choi J.S. (2004). Inhibitory phlorotannins from the edible brown alga *Ecklonia stolonifera* on total reactive oxygen species (ROS) generation. Arch. Pharm. Res..

[22] Seong S.H., Paudel P., Choi J.-W., Ahn D.H., Nam T.-J., Jung H.A., Choi J.S. (2019). Probing multi-target action of phlorotannins as new monoamine oxidase inhibitors and dopaminergic receptor modulators with the potential for treatment of neuronal disorders. Mar. Drugs.

[23] Paricharak S., Cortés-Ciriano I., IJzerman A.P., Malliavin T.E., Bender A. (2015). Proteochemometric modelling coupled to in silico target prediction: An integrated approach for the simultaneous prediction of polypharmacology and binding affinity/potency of small molecules. J. Cheminform..

[24] Burda S., Oleszek W. (2001). Antioxidant and antiradical activities of flavonoids. J. Agric. Food Chem..

[25] Dugas A.J., Castañeda-Acosta J., Bonin G.C., Price K.L., Fischer N.H., Winston G.W. (2000). Evaluation of the total peroxyl radical-scavenging capacity of flavonoids: Structure- activity relationships. J. Nat. Prod..

[26] Shibata T., Ishimaru K., Kawaguchi S., Yoshikawa H., Hama Y., Borowitzka M.A., Critchley A.T., Kraan S., Peters A., Sjøtun K., Notoya M. (2007). Antioxidant activities of phlorotannins isolated from Japanese Laminariaceae. Nineteenth International Seaweed Symposium.

[27] Borroto-Escuela D.O., Romero-Fernandez W., Tarakanov A.O., Gómez-Soler M., Corrales F., Marcellino D., Narvaez M., Frankowska M., Flajolet M., Heintz N. (2010). Characterization of the A2AR–D2R interface: Focus on the role of the C-terminal tail and the transmembrane helices. Biochem. Biophys. Res. Commun..

[28] Fuxe K., Agnati L.F., Jacobsen K., Hillion J., Canals M., Torvinen M., Tinner-Staines B., Staines W., Rosin D., Terasmaa A. (2003). Receptor heteromerization in adenosine A2A receptor signaling: Relevance for striatal function and Parkinson’s disease. Neurology.

[29] Ferre S., Von Euler G., Johansson B., Fredholm B.B., Fuxe K. (1991). Stimulation of high-affinity adenosine A2 receptors decreases the affinity of dopamine D2 receptors in rat striatal membranes. Proc. Natl. Acad. Sci. USA.

[30] Yamada K., Kobayashi M., Kanda T. (2014). Involvement of Adenosine A2A Receptors in Depression and Anxiety. Int. Rev. Neurobiol..

[31] Jaakola V.-P., Griffith M.T., Hanson M.A., Cherezov V., Chien E.Y., Lane J.R., Ijzerman A.P., Stevens R.C. (2008). The 2.6 angstrom crystal structure of a human A2A adenosine receptor bound to an antagonist. Science.

[32] Kang M.C., Cha S.H., Wijesinghe W.A., Kang S.M., Lee S.H., Kim E.A., Song C.B., Jeon Y.J. (2013). Protective effect of marine algae phlorotannins against AAPH-induced oxidative stress in zebrafish embryo. Food Chem..

[33] Cha S.-H., Heo S.-J., Jeon Y.-J., Park S.M. (2016). Dieckol, an edible seaweed polyphenol, retards rotenone-induced neurotoxicity and α-synuclein aggregation in human dopaminergic neuronal cells. RSC Adv..

[34] Cui Y., Park J.Y., Wu J., Lee J.H., Yang Y.S., Kang M.S., Jung S.C., Park J.M., Yoo E.S., Kim S.H. (2015). Dieckol Attenuates Microglia-mediated Neuronal Cell Death via ERK, Akt and NADPH Oxidase-mediated Pathways. Korean J. Physiol. Pharmacol..

[35] Kim J.-J., Kang Y.-J., Shin S.-A., Bak D.-H., Lee J.W., Lee K.B., Yoo Y.C., Kim D.-K., Lee B.H., Kim D.W. (2016). Phlorofucofuroeckol improves glutamate-induced neurotoxicity through modulation of oxidative stress-mediated mitochondrial dysfunction in PC12 cells. PLoS ONE.

[36] Gaidin S.G., Turovskaya M.V., Mal’tseva V.N., Zinchenko V.P., Blinova E.V., Turovsky E.A. (2019). A Complex Neuroprotective Effect of Alpha-2-Adrenergic Receptor Agonists in a Model of Cerebral Ischemia–Reoxygenation In Vitro. Biochemistry.

[37] Bücheler M.M., Hadamek K., Hein L. (2002). Two α2-adrenergic receptor subtypes, α2A and α2C, inhibit transmitter release in the brain of gene-targeted mice. Neuroscience.

[38] Scheinin M., Sallinen J., Haapalinna A. (2001). Evaluation of the α2C-adrenoceptor as a neuropsychiatric drug target: Studies in transgenic mouse models. Life Sci..

[39] Fairbanks C.A., Stone L.S., Kitto K.F., Nguyen H.O., Posthumus I.J., Wilcox G.L. (2002). α2C-Adrenergic receptors mediate spinal analgesia and adrenergic-opioid synergy. J. Pharmacol. Exp. Ther..

[40] Duflo F., Li X., Bantel C., Pancaro C., Vincler M., Eisenach J.C. (2002). Peripheral Nerve Injury Alters the α2Adrenoceptor Subtype Activated by Clonidine for Analgesia. Anesthesiology.

[41] Quaglia W., Del Bello F., Giannella M., Piergentili A., Pigini M. (2011). α2C-adrenoceptor modulators: A patent review. Expert. Opin. Ther. Pat..

[42] Chilmonczyk Z., Bojarski A.J., Pilc A., Sylte I. (2015). Functional selectivity and antidepressant activity of serotonin 1A receptor ligands. Int. J. Mol. Sci..

[43] Simon N.G., Guillon C., Fabio K., Heindel N.D., Lu S.-f., Miller M., Ferris C.F., Brownstein M.J., Garripa C., Koppel G.A. (2008). Vasopressin antagonists as anxiolytics and antidepressants: Recent developments. Recent Pat. CNS Drug Discov..

[44] Narayen G., Mandal S.N. (2012). Vasopressin receptor antagonists and their role in clinical medicine. Indian J. Endocrinol. Metab..

[45] Liu X., Luo G., Jiang J., Ma T., Lin X., Jiang L., Cheng J., Tao R. (2016). Signaling through hepatocyte vasopressin receptor 1 protects mouse liver from ischemia-reperfusion injury. Oncotarget.

[46] Glass M., Faull R., Dragunow M. (1997). Cannabinoid receptors in the human brain: A detailed anatomical and quantitative autoradiographic study in the fetal, neonatal and adult human brain. Neuroscience.

[47] Kano M., Ohno-Shosaku T., Hashimotodani Y., Uchigashima M., Watanabe M. (2009). Endocannabinoid-mediated control of synaptic transmission. Physiol. Rev..

[48] Xu X., Liu Y., Huang S., Liu G., Xie C., Zhou J., Fan W., Li Q., Wang Q., Zhong D. (2006). Overexpression of cannabinoid receptors CB1 and CB2 correlates with improved prognosis of patients with hepatocellular carcinoma. Cancer Genet. Cytogenet..

[49] Qamri Z., Preet A., Nasser M.W., Bass C.E., Leone G., Barsky S.H., Ganju R.K. (2009). Synthetic cannabinoid receptor agonists inhibit tumor growth and metastasis of breast cancer. Mol. Cancer Ther..

[50] Ogden S.B., Malamas M.S., Makriyannis A., Eckel L.A. (2019). The novel cannabinoid CB(1) receptor agonist AM11101 increases food intake in female rats. Br. J. Pharmacol..

[51] Kim A.-R., Lee M.-S., Choi J.-W., Utsuki T., Kim J.-I., Jang B.-C., Kim H.-R. (2013). Phlorofucofuroeckol A suppresses expression of inducible nitric oxide synthase, cyclooxygenase-2, and pro-inflammatory cytokines via inhibition of nuclear factor-κB, c-Jun NH 2-terminal kinases, and Akt in microglial cells. Inflammation.

[52] Eo H.J., Kwon T.H., Park G.H., Song H.M., Lee S.J., Park N.H., Jeong J.B. (2016). In Vitro Anticancer Activity of Phlorofucofuroeckol A via Upregulation of Activating Transcription Factor 3 against Human Colorectal Cancer Cells. Mar. Drugs.

[53] D’Antona A.M., Ahn K.H., Kendall D.A. (2006). Mutations of CB1 T210 produce active and inactive receptor forms: Correlations with ligand affinity, receptor stability, and cellular localization. Biochemistry.

[54] Römpler H., Yu H.-T., Arnold A., Orth A., Schöneberg T. (2006). Functional consequences of naturally occurring DRY motif variants in the mammalian chemoattractant receptor GPR33. Genomics.

[55] Krishna Kumar K., Shalev-Benami M., Robertson M.J., Hu H., Banister S.D., Hollingsworth S.A., Latorraca N.R., Kato H.E., Hilger D., Maeda S. (2019). Structure of a Signaling Cannabinoid Receptor 1-G Protein Complex. Cell.

[56] Lin L.S., Ha S., Ball R.G., Tsou N.N., Castonguay L.A., Doss G.A., Fong T.M., Shen C.-P., Xiao J.C., Goulet M.T. (2008). Conformational Analysis and Receptor Docking of N-[(1S,2S)-3-(4-Chlorophenyl)-2-(3-cyanophenyl)-1-methylpropyl]-2-methyl-2-{[5-(trifluoromethyl)pyridin-2-yl]oxy}propanamide (Taranabant, MK-0364), a Novel, Acyclic Cannabinoid-1 Receptor Inverse Agonist. J. Med. Chem..

[57] Turton M., O’shea D., Gunn I., Beak S., Edwards C., Meeran K., Choi S., Taylor G., Heath M., Lambert P. (1996). A role for glucagon-like peptide-1 in the central regulation of feeding. Nature.

[58] Drucker D.J., Nauck M.A. (2006). The incretin system: Glucagon-like peptide-1 receptor agonists and dipeptidyl peptidase-4 inhibitors in type 2 diabetes. Lancet.

[59] Kim E.-A., Lee S.-H., Lee J.-H., Kang N., Oh J.-Y., Ahn G., Ko S.C., Fernando S.P., Kim S.-Y., Park S.-J. (2016). A marine algal polyphenol, dieckol, attenuates blood glucose levels by Akt pathway in alloxan induced hyperglycemia zebrafish model. RSC Adv..

[60] Paudel P., Seong S.H., Fauzi F.M., Bender A., Jung H.A., Choi J.S. (2020). Establishing GPCR targets of hMAO active anthraquinones from *Cassia obtusifolia* Linn seeds using in silico and in vitro methods. ACS Omega.

[61] Mohd Fauzi F., John C.M., Karunanidhi A., Mussa H.Y., Ramasamy R., Adam A., Bender A. (2017). Understanding the mode-of-action of *Cassia auriculata* via in silico and in vivo studies towards validating it as a long term therapy for type II diabetes. J. Ethnopharmacol..

[62] Paudel P., Seong S.H., Jung H.A., Choi J.S. (2019). Characterizing fucoxanthin as a selective dopamine D3/D4 receptor agonist: Relevance to Parkinson’s disease. Chem. Biol. Interact..

[63] Goodsell D.S., Morris G.M., Olson A.J. (1996). Automated docking of flexible ligands: Applications of AutoDock. J. Mol. Recognit..

[64] Granier S., Manglik A., Kruse A.C., Kobilka T.S., Thian F.S., Weis W.I., Kobilka B.K. (2012). Structure of the δ-opioid receptor bound to naltrindole. Nature.

[65] Shao Z., Yan W., Chapman K., Ramesh K., Ferrell A.J., Yin J., Wang X., Xu Q., Rosenbaum D.M. (2019). Structure of an allosteric modulator bound to the CB1 cannabinoid receptor. Nat. Chem. Biol..

[66] Song G., Yang D., Wang Y., de Graaf C., Zhou Q., Jiang S., Liu K., Cai X., Dai A., Lin G. (2017). Human GLP-1 receptor transmembrane domain structure in complex with allosteric modulators. Nature.

