# Supplementary material for: In Vitro and In Silico Characterization of G-Protein Coupled Receptor (GPCR) Targets of Phlorofucofuroeckol-A and Dieckol
Source: Mar Drugs. 2021 Jun 4;19(6):326. doi: 10.3390/md19060326 (PMC8228075; doi:10.3390/md19060326)
Supplement: Supplementary file 1 [file marinedrugs-19-00326-s001.zip › marinedrugs-1235081-supplementary.pdf]

# In Vitro and In Silico Characterization of G-protein Coupled Receptor (GPCR) Targets of Phlorofucofuroeckol-A and Dieckol

**Pradeep Paudel [1,2,†], Su Hui Seong [1,3,†], Se Eun Park [1,4], Jong Hoon Ryu [5], Hyun Ah Jung [6,*], and Jae Sue Choi[1,*]**

[1] Department of Food and Life Science, Pukyong National University, Busan 48513, Republic of Korea; ppradeep@olemiss.edu (P.P.); shseong@hnibr.re.kr (S.H.S.); gogo1685@mail.ulsan.ac.kr (S.E.P.)
[2] National Center for Natural Products Research, Research Institute of Pharmaceutical Sciences, The University of Mississippi, Oxford, MS 38677, USA
[3] Natural Products Research Division, Honam National Institute of Biological Resource, Mokpo 58762, Republic of Korea
[4] Department of Biomedical Science, Asan Medical Institute of Convergence Science and Technology, Seoul 05505, Republic of Korea
[5] Department of Life and Nanopharmaceutical Science, Kyung Hee University, Seoul 02447, Republic of Korea; jhryu63@khu.ac.kr (J.H.R.)
[6] Department of Food Science and Human Nutrition, Jeonbuk National University, Jeonju 54896, Republic of Korea
* Correspondence: jungha@jbnu.ac.kr (H.A.J.); choijs@pknu.ac.kr (J.S.C.); Tel.: +82-63-270-4882 (H.A.J.); +82-51-629-7547 (J.S.C.)
† These authors contributed equally to this work.

**Table S1.** Hydrogen bonding interaction residues between ligand-GPCRs ($hA_{2A}R$, $h\alpha_{2C}AR$, and hDOP).

| Compounds | Target GPCRs (PDB ID) | | | | |
|---|---|---|---|---|---|
| | **$hA_{2A}R$ (3eml)** | **Binding score (kcal/mol)** | **$h\alpha_{2C}AR$ (6kuw)** | **Binding score (kcal/mol)** | **hδ-OPR (4ej4)** |
| Standard agonist [a] | Ser277, Glu169, Asn253 | −5.56 | Asp131 (Salt bridge, H-bond), Ser218, Val132, Thr136, Ser214 | −6.63 | Asp128 (Salt bridge), Ile277, His278, Lys214 |
| Standard antagonist [b] | Asn253, Glu169 | −8.93 | Asp131 (Salt bridge), Ser218 | −11.68 | Asp128 (Salt bridge, H-bond) |
| Dieckol | Ile80, Asp170, | −9.18 | Asn111, Ser108, Cys202, Asp206, Gly203 | −7.13 | Asp128, Met132, Cys198 |
| Phlorofucofuroeckol-A | His278, Ala59, Ala81, Ser67 | −9.03 | Val414, Asp131, Ser401, Gln413 | −9.11 | Leu200, Lys214, Ile304, Asp128 |

[a] 5′-N-ethylcarboxamidoadenosine (NECA) for $hA_{2A}R$, epinephrine for $h\alpha_{2C}AR$, and DPI-287 for hDOP.

[b] ZM241385 for $hA_{2A}R$, RS-79948 for $h\alpha_{2C}AR$, and naltrindole for hDOP.

**Table S2.** Hydrophobic and electrostatic interaction residues between ligand-GPCRs (h$A_{2A}$R, h$\alpha_{2C}$AR, and hDOP).

| Compounds | Target GPCRs (PDB ID) | | |
|---|---|---|---|
| | **h$A_{2A}$R (3eml)** | **h$\alpha_{2C}$AR (6kuw)** | **hδ-OPR (4ej4)** |
| Standard agonist [a] | Pi-Pi: Phe168<br>Pi-Sigma: His250<br>Pi-Alkyl: Leu249, Met270 | Pi-Pi: Phe399 (T-shaped)<br>Pi-Sigma: Val132<br>Pi-Alkyl: Cys135 | Pi-Pi: Tyr308<br>Pi-Sigma: Val281, Tyr129<br>Pi-Sulfur: Met132<br>Pi-Alkyl: Val281, Ile277, Ile304, Val217<br>Alkyl: Met132, Met199, Leu125 |
| Standard antagonist [b] | Pi-Pi: Phe168<br>Pi-Anion: Glu169<br>Pi-Sigma: Leu249<br>Pi-Sulfur: Met270<br>Pi-Alkyl: Met177 | Pi-Sigma: Phe423, Tyr402<br>Pi-Sulfur: Phe398, Phe419<br>Pi-Alkyl: Phe423 | Pi-Pi: His278 (T-shaped), Trp284 (T-shaped)<br>Pi-Sigma: Val281 / Pi-Sulfur: Met132<br>Pi-Alkyl: Val281, Trp284, Trp274, Tyr308, Ile277 |
| Dieckol | Pi-Pi: Phe168<br>Pi-Sigma: Leu249, Ile274<br>Pi-Sulfur: Met270 / Pi-Anion: Asp170<br>Amide-Pi stacked: Phe62<br>Pi-Alkyl: Leu167, Leu267, Val84, Ala63, Ile66, Val84, Met270 | Pi-Pi: Phe419, Phe423, Tyr405<br>Pi-Alkyl: Leu204 | Pi-Sigma: Ile304, Val281<br>Pi-Sulfur: Cys198<br>Pi-Anion: Asp128 / Pi-Cation: Lys108<br>Pi-Alkyl: Ile277, Val197, Lys108, Met132<br>Sulfur-O: Met132 |
| Phlorofucofuroeckol-A | Pi-Pi: Phe168<br>Pi-Sigma: Val84, Leu249, Ile274<br>Pi-Alkyl: Ile274, Ile66, Ala63, Leu167 | Pi-Pi: Phe419, Tyr405 (T-shaped)<br>Pi-Sigma: Gly203<br>Pi-Anion: Asp131<br>Pi-Alkyl: Leu204<br>Pi-Lone pair: Tyr405 | Pi-Pi: Tyr308<br>Pi-Anion: Asp128, Asp210<br>Pi-Lone Pair: Tyr129<br>Pi-Alkyl: Leu200 |

[a] 5′-N-ethylcarboxamidoadenosine (NECA) for h$A_{2A}$R, epinephrine for h$\alpha_{2C}$AR, and DPI-287 for hDOP.

[b] ZM241385 for h$A_{2A}$R, RS-79948 for h$\alpha_{2C}$AR, and naltrindole for hDOP.

**Table S3**. Hydrogen, halogen, or electrostatic bonding interaction residues between ligand-GPCRs (h$CB_1R$ and hGLP-1).

| Compounds | Target GPCRs | | | |
|---|---|---|---|---|
| | **h$CB_1R$** | **Binding score (kcal/mol)** | **hGLP-1** | **Binding score (kcal/mol)** |
| Standard agonist [a] | Ser173, Ser383, Ile267 | −10.50 | Gln37, Lys197, Arg380 (Attractive charge) | −14.10 |
| Standard antagonist [b] | Ser173, Ser383, Ser173 (F), Phe189 (F), Lys192 (F) | −9.34 | Thr355, Ser352, Asn406 | −10.06 |
| Dieckol | *ND* | *ND* | Ser352, Ile328, Leu354 | −5.46 |
| Phlorofucofuroeckol-A | Ser173, His178, Met363 | −6.40 | Ile328, Thr355, Val405, Ser352 | −6.73 |

[a] CP 55940 (ligand for 6kqi) for h$CB_1R$ and PF-06882961 (ligand for 6x1a) for hGLP-1.

[b] Taranabant (ligand for 5u09) for h$CB_1R$, and NNC0640 (ligand for 5vex) for hGLP-1.

ND Not determined.

**Table S4.** Hydrophobic interaction residues between ligand-GPCRs ($hCB_1R$ and hGLP-1).

| Compounds | Target GPCRs | |
|---|---|---|
| | **$hCB_1R$** | **hGLP-1** |
| Standard agonist [a] | Pi-Pi: Phe170 (T-shaped), Phe268 (T-shaped)<br>Pi-Alkyl: Phe170, Phe174, Phe177, Tyr275, Trp279, Trp356<br>Alkyl: Leu276, Val196, Leu359 | Pi-Pi: Trp33, Phe230 (T-shaped)<br>Pi-Sigma/Alkyl: Leu217, Trp203, Phe230, Phe381, Lys197, Leu32, Val36<br>Alkyl: Cys296 |
| Standard antagonist [b] | Pi-Pi: Phe170, Phe268, Trp279 (T-shaped)<br>Pi-Sigma/Sulfur/Alkyl: Thr197, Cys386, Trp279, Phe174, His178, Phe189, Phe268, Phe379, Val196, Leu193, Met363<br>Alkyl: Ile267, Lys192, Leu193, Leu276, Met363 | Pi-Sigma/Alkyl: Arg348, Thr355, Leu354, Lys351, Val405 |
| Dieckol | ND | Pi-Cation: Lys351<br>Pi-Sigma: Ile328<br>Pi-Alkyl: Ile328, Lys351, Val405, Arg348, Leu354, Val331, |
| Phlorofucofuroeckol-A | Pi-Pi: Phe177, Trp279 (T-shaped), Phe268 (T-shaped)<br>Pi-Alkyl: Val196, Leu193, Met363 | Pi-Cation: Lys351<br>Pi-Sigma: Leu354<br>Pi-Alkyl: Leu354, Lys351, Ile328, Val331, Ala350, Lys351, Val405 |

[a] CP 55940 (ligand for 6kqi) for $hCB_1R$ and PF-06882961 (ligand for 6x1a) for hGLP-1.
[b] Taranabant (ligand for 5u09) for $hCB_1R$, and NNC0640 (ligand for 5vex) for hGLP-1.
ND Not determined.

**Table S5**. Hydrogen bonding interaction residues between ligand-GPCRs ($hV_{1A}R$ and $h5\text{-}HT_{1A}R$).

| Compounds | Target GPCRs | | | |
|---|---|---|---|---|
| | **$hV_{1A}R$** | **Binding score (kcal/mol)** | **$h5\text{-}HT_{1A}R$** | **Binding score (kcal/mol)** |
| Standard agonist [a] | Asp202 (Salt-bridge), Glu54, Asp112, Ile330 | –9.14 | Asp116 (Salt bridge), Thr200, Thr121 | –6.77 |
| Standard antagonist [b] | Gln131, Gln108, Lys128 | –8.98 | Asp116 (Salt bridge), Asn386 | –10.31 |
| Dieckol | Gln131, Ala334, Asp112 | –8.33 | Asp116, Thr200, Ser190, Asn386, Tyr96 | –10.38 |
| Phlorofucofuroeckol-A | Ser338, Cys203, Met135, Glu54, Ala101 | –9.34 | Thr188, Glu372, Tyr96, Asn386 | –7.45 |

[a] AVP for $hV_{1A}R$ and serotonin for $h5\text{-}HT_{1A}R$.

[b] SR49059 for $hV_{1A}R$ and WAY 100635 for $h5\text{-}HT_{1A}R$.

**Table S6.** Hydrophobic and electrostatic interaction residues between ligand-GPCRs (h$V_{1A}$R and h5-$HT_{1A}$R).

| Compounds | Target GPCRs | |
|---|---|---|
| | **h$V_{1A}$R** | **h5-$HT_{1A}$R** |
| Standard agonist[a] | Pi-Cation: Trp204<br>Pi-Alkyl: Ala101, Ala334, Val132, Met135<br>Alkyl: Ile330 | Pi-Pi: Phe362 (T-shaped)<br>Pi-Cation: Phe361<br>Pi-Sulfur: Cys120<br>Pi-Alkyl: Val117, Ala203 |
| Standard antagonist[b] | Pi-Pi: Trp204 (T-shaped), Phe307 (T-shaped)<br>Pi-Sigma: Phe307<br>Pi-Alkyl: Trp204, Ala334, Ala205<br>Alkyl: Val132, Met135 | Pi-Pi: Phe362 (T-shaped)<br>Pi-Sulfur: Cys120<br>Pi-Lone Pair: Asn386<br>Pi-Alkyl: Phe112, Ala203<br>Alkyl: Ile113 |
| Dieckol | Pi-Pi: Trp204<br>Pi-Cation: Lys128<br>Pi-Sulfur: Met135<br>Pi-Alkyl: Ala101, Ala334, Lys128, Val132 | Pi-Sigma: Thr188, Ile19, Thr196<br>Pi-Alkyl: Ile189, Val117 |
| Phlorofucofuroeckol-A | Pi-Pi: Phe189 (T-shaped), Phe307 (T-shaped)<br>Pi-cation: Lys128<br>Pi-Sulfur: Met220<br>Pi-Alkyl: Val132, Val100, Ala101, Met135, Ala334, Ala205, Val105 | Pi-Pi: Phe361<br>Pi-Sigma: Thr188<br>Pi-Alkyl: Lys191<br>Pi-Anion: Asp116<br>Pi-Cation: Lys191 |

[a] AVP for h$V_{1A}$R and serotonin for h5-$HT_{1A}$R.

[b] SR49059 for h$V_{1A}$R and WAY 100635 for h5-$HT_{1A}$R.
